# The Effect of Fenugreek in Type 2 Diabetes and Prediabetes: A Systematic Review and Meta-Analysis of Randomized Controlled Trials

**DOI:** 10.3390/ijms241813999

**Published:** 2023-09-12

**Authors:** Jiwon Kim, Woojeong Noh, Ahrim Kim, Yoomin Choi, Young-Sik Kim

**Affiliations:** 1 College of Korean Medicine, Woosuk University, Jeonju 54986, Republic of Korea; kjw524s@naver.com (J.K.); woojeong1108@naver.com (W.N.); zlwmdkfla@naver.com (A.K.); 2 Department of Acupuncture & Moxibustion Medicine, College of Korean Medicine, Woosuk University, Jeonju 54986, Republic of Korea; yoomin@woosuk.ac.kr; 3 Department of Herbology, College of Korean Medicine, Woosuk University, Jeonju 54986, Republic of Korea

**Keywords:** fenugreek, trigonella, diabetes, T2DM, systematic review, meta-analysis

## Abstract

Fenugreek is a widely used herbal medicine as a complementary therapy for diabetes mellitus. Lots of clinical trials have proved its beneficial effect on glycemic control parameters and lipid profiles. Thus, we conducted a systematic review and meta-analysis to evaluate the effectiveness and safety of fenugreek as a treatment for type 2 diabetes mellitus. We searched PubMed, Embase, Cochrane, China Knowledge Resource Integrated Database (CNKI), Korean studies Information Service System (KISS), Research Information Sharing Service (RISS), and ScienceON to select RCTs which used fenugreek targeting hyperglycemia with a control group. We used either a random effect model or a fixed model in a meta-analysis of Fasting blood glucose (FBG), 2 h plasma glucose during a 75 g oral glucose tolerance test (OGTT) (2-hPG), homeostatic model assessment for insulin resistance (HOMA-IR), glycosylated hemoglobin (HbA1c)/total cholesterol (TC), triglyceride (TG), high density lipoprotein (HDL-C), low density lipoprotein (LDL-C), body mass index (BMI). After screening, a total of 10 studies (706 participants) remained. Fenugreek significantly reduced FBG, 2-hPG, and HbA1c, but it did not significantly decrease HOMA-IR. Moreover, it significantly improved TC, TG, and HDL-C, while there were no significant differences in LDL-C and BMI. Hepatic or renal toxicity was not observed, and there were no severe adverse events associated with fenugreek despite mild gastrointestinal side effects in some studies. In conclusion, fenugreek improves overall glycemic control parameters and lipid profile safely.

## 1. Introduction

According to the IDF (International Diabetes Federation) [1], one in ten (10.5%) adults around the world currently live with diabetes mellitus. The total number is predicted to rise to 783 million (12.2%) by 2045. Globally, over 90% of people with diabetes have type 2 diabetes mellitus (T2DM). The rise in the number of people with T2DM is driven by a complex interplay of socio-economic, demographic, environmental, and genetic factors. Key contributors include urbanization, an aging population, a decrease in physical activity, and an increasing prevalence of overweight and obesity. Furthermore, 10.6% of adults worldwide have impaired glucose tolerance (IGT), often referred to as prediabetes, placing them at high risk of developing T2DM [2]. Thus, it is necessary and important to manage not only T2DM but also prediabetes.

Diabetes mellitus is mainly associated with various values such as fasting blood glucose (FBG), 2 h plasma glucose (2-hPG) during a 75 g oral glucose tolerance test (OGTT), homeostatic model assessment for insulin resistance (HOMA-IR), and glycosylated hemoglobin (HbA1c). Body mass index (BMI) and lipid profile, including total cholesterol (TC), triglyceride (TG), high density lipoprotein (HDL-C), and low density lipoprotein (LDL-C), are also associated with diabetes mellitus. Problems with the lipid profile can cause diabetic complications such as diabetic peripheral neuropathy (DPN) [3] and diabetic retinopathy. Moreover, the coexistence of diabetes with dyslipidemia further increases the risk of cardiovascular disease [4]. Likewise, higher BMI values are associated with an elevated risk of developing diabetic complications [5].

Commonly used anti-diabetic drugs include sulfonylureas, metformin, and sodium-glucose cotransporter 2 (SGLT-2) inhibitors. However, sulfonylureas are associated with hypoglycemia and weight gain; metformin may lead to gastrointestinal disturbances such as diarrhea, nausea, and dyspepsia, as well as the risk of lactic acidosis; while SGLT-2 inhibitors can potentially result in urogenital infections. Additionally, the high cost of these medications often makes patients reluctant to use them. As a result, extensive research has been conducted to explore the potential of natural substances in plants for the treatment of diabetes mellitus and the control of diabetic complications [6]. Complementary and alternative medicine (CAM) has also been extensively studied in diabetes mellitus [7,8,9]. Fenugreek is one of the herbal medicines used in these treatments [10].

*Trigonella foenum-graecum*, an annual plant of the Fabaceae family, is also known as fenugreek or 葫蘆巴. Fenugreek contains abundant active compounds, including galactomannan [11], saponins, diosgenin, and 4-hydroxyisoleucine (4-OH-Ile) [12], which possess therapeutic properties for humans and animals. These active compounds give fenugreek various benefits such as being anti-inflammatory, anti-carcinogenic, hypoglycemic, anti-hypertensive, immunomodulatory, hypocholesterolemic, neuroprotective, antioxidant, reproductive, gastroprotective, galactogogue, and hepatoprotective [13].

Currently, as the prevalence of diabetes increases worldwide, the applicability of fenugreek for diabetes mellitus has been studied in Asia [14], India [15], the Middle East [16], and North America [17]. A previous systematic review on the improvement through glucose tolerance of medicinal food revealed that fenugreek has more significant effects on FBG and HOMA-IR than other medicinal foods for impaired glucose tolerance (acacia polyphenol, cinnamon, curcumin, Korean red ginseng, *Elaeis guineensis* leaf extract, flaxseed, *Panax ginseng* hydrolyzed extract, *Ginkgo biloba*, and *Salacia reticulata* leaves extract [18]). Additionally, a previous meta-analysis study reported that fenugreek improves TC, TG, LDL, and HDL in individuals with T2DM [19]. Therefore, fenugreek is suggested to be an effective complementary and alternative medicine for controlling the blood glucose and lipid profile in individuals with T2DM and prediabetes. Although systematic reviews and meta-analyses on the anti-diabetic and hypocholesterolemic effects of fenugreek have been reported, there is only one study published by Gong et al., 2016 [20], regarding the effect of fenugreek on diabetic hyperglycemia and hyperlipidemia. However, within the last 7 years after Gong’s study, a number of clinical trials have been conducted. Therefore, our study aimed to update the latest version of systematic review and meta-analysis on the effectiveness and safety of fenugreek in individuals with T2DM and prediabetes.

## 2. Materials and Methods

We conducted this systematic review and meta-analysis according to the Preferred Reporting Items for Systematic Review and Meta-Analysis (PRISMA) checklist [21]. The protocol of this study was registered in the International Prospective Register of Systematic Reviews (PROSPERO, CRD42022315715).

### 2.1. Data Sources and Searches

We searched six electronic databases to find relevant randomized controlled trials in July 2023. The electronic databases were PubMed, Embase, Cochrane, China Knowledge Resource Integrated Database (CNKI), Korean studies Information Service System (KISS), Research Information Sharing Service (RISS), and ScienceON. We referred to Korean and Chinese pharmacopoeia to find the exact name of fenugreek [22,23]. Search terms are indicated in Table 1.

### 2.2. Study Selection

Our study was based on clinical trials, so non-clinical trials such as animal, in vitro, review, and cross-sectional studies were excluded. In addition, among clinical trials, non-RCTs were also excluded. Our participant criteria were established by the criteria for diagnosing prediabetes in the American Diabetes Association (ADA) [24]. We indicated the inclusion and exclusion criteria in Table 2 and Table 3. Selection of RCTs was performed by the established criteria. 

### 2.3. Data Extraction

The process of data extraction was performed by two reviewers (JW, WJ) based on lead author, publication year, location of the study, study design, duration, characteristics of participants (age, gender, hypoglycemic drugs intake), sample size in each group, intervention (daily dose, type), controls (daily dose, type), additional therapy (if applicable), and the primary outcomes of each group and adverse events. If each parameter unit of primary outcomes was different, we matched them to the most commonly used units.

### 2.4. Quality Assessment

We used the Revised Cochrane risk-of-bias tool for randomized trials (ROB 2.0) to assess the quality of the included studies. This tool is made up of the following domains: (1) randomization process; (2) deviations from the intended interventions; (3) missing outcomes; (4) measurement of the outcome; and (5) selection of reported results. At first, two reviewers (JW, WJ) performed the quality assessment of the included studies independently. In case of inconsistencies, all reviewers (JW, WJ, AR) reassessed them through discussion. If disagreements continued, we made a final decision with the assistant professor (YS).

### 2.5. Data Synthesis and Meta-Analysis

We calculated the mean differences (MDs) within each group (i.e., post-treatment values minus pre-treatment values) and the standard deviation (SD) changes in outcomes to compare the changes before and after treatment. We calculated SD using the following formula: SD = sqrt [(SD_pre-treatment_)^2^ + (SD_post-treatment_)^2^ − (2R × SD_pre-treatment_ × SD_post-treatment_)], where R, a correlation coefficient, was equal to 0.5 [25]. To indicate the effect sizes of continuous outcomes, we used the MD with corresponding 95% confidence intervals (CIs). We employed Cochrane Q statistics and I^2^ statistics to estimate statistical heterogeneity. The I^2^ value indicates the percentage of variability in effect estimates that is due to heterogeneity rather than sampling error [26]. Traditionally, as suggested by Jack Bowden and Jayne F Tierney, a high level of inconsistency is indicated by an I^2^ value of over 75%; a moderate inconsistency is indicated by an I^2^ value of above 50%; and low inconsistency is suggested by an I^2^ value of below 25% [27]. We basically applied a fixed effects model, but if the I^2^ value was over 75%, we used a random effects model. Also, if there was considerable heterogeneity in the results (I^2^ is over 50%), we conducted a sensitivity analysis by omitting one study at once. Funnel-plot-analysis and Egger’s tests were used to examine potential publication bias. Subgroup analysis was conducted to find differences according to treatment method (combination therapy vs. monotherapy) and intervention type (extracts vs. raw material). At first, we had planned to perform a subgroup analysis according to treatment method and different stage of disease progression (diabetes, prediabetes, and intermediate hyperglycemia) as mentioned in our protocol. However, all participants of remained studies after screening were T2DM and prediabetics. Moreover, the number of participants was insufficient for subgroup analysis because only one study [28] targeted prediabetes. Thus, subgroup analysis of different stage of disease progression could not be performed as planned, and we changed it into intervention type, which is thought to have an effect on the results. All data analyses were conducted using the Cochrane Collaboration’s software program Review Manager (RevMan) version 5.4.1. and R 4.2.1 The *p* value of below 0.05 was considered statistically significant.

## 3. Results

### 3.1. Study Selection

**Figure 1:**
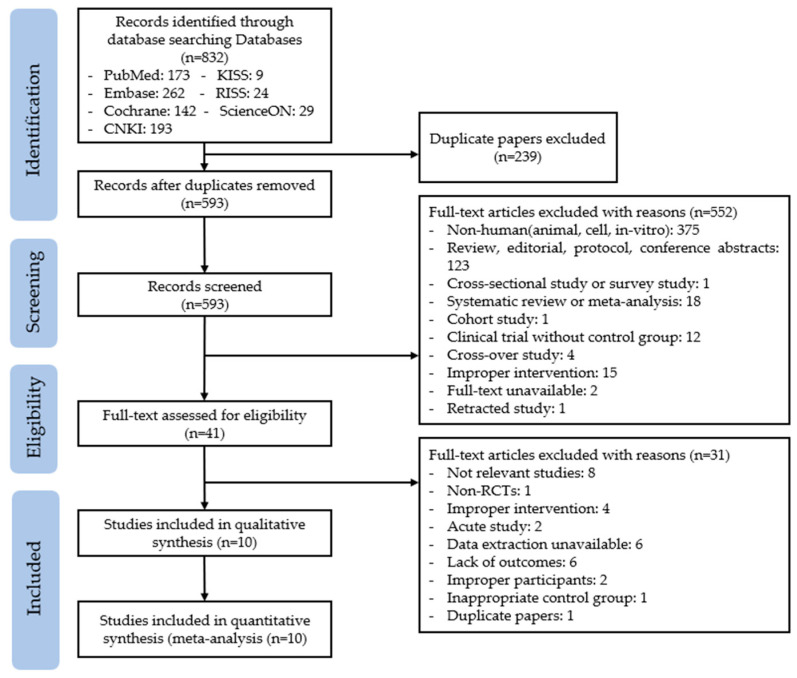
PRISMA flow diagram of study selection.

The information of the screening process is illustrated in Figure 1. We identified 832 articles after the initial database searches. All articles were imported into Endnote, and 239 results were checked for duplicates. After removing the duplicates, 593 articles remained for primary screening based on the title, abstract, and keywords. In total, 552 articles were screened by our inclusion and exclusion criteria, so secondary screening was conducted with 41 articles by reading the full-text in depth. Finally, 10 articles were included in the quality assessment and meta-analysis steps. 

### 3.2. Characteristics of Included Studies

A total of 706 participants were included in our study, with ages ranging from 25 to 70 years; 49% of the participants were male. While one study [29] was performed on only females, the rest were performed on both genders. Two of the trials of the included studies [29,30] were conducted with four groups, but for our analysis, we only selected the groups that used fenugreek and the placebo, without any additional treatment to compare the effect of only fenugreek. Initially, our inclusion criteria targeted individuals with high glucose levels, but after the screening process, only participants with type 2 diabetes mellitus (T2DM) remained. The duration of these studies ranged from 8 to 16 weeks. The type of intervention was divided into extracts (five studies [11,28,31,32,33]) and the raw material group (five studies [29,30,34,35,36]). One study of the non-extracts group [34] did not report about intervention type exactly, but mentioned a large dosage; we estimated that this study used the whole seed of fenugreek. The others of raw material group used powdered fenugreek seeds. In five studies [11,33,34,35,36], the subjects received the dietary management. Two studies [11,34] added exercise training. All included studies were RCTs in a parallel design, and they consisted of six double blind, one single blind, one triple blind and two open-label trials. The studies were conducted in India, China, Pakistan, Iran, and Australia. The characteristics of included studies are indicated in Table 4. 

### 3.3. Effect of Fenugreek Supplementation on Glycemic Control Parameters

#### 3.3.1. Fasting Blood Glucose

**Figure 2:**
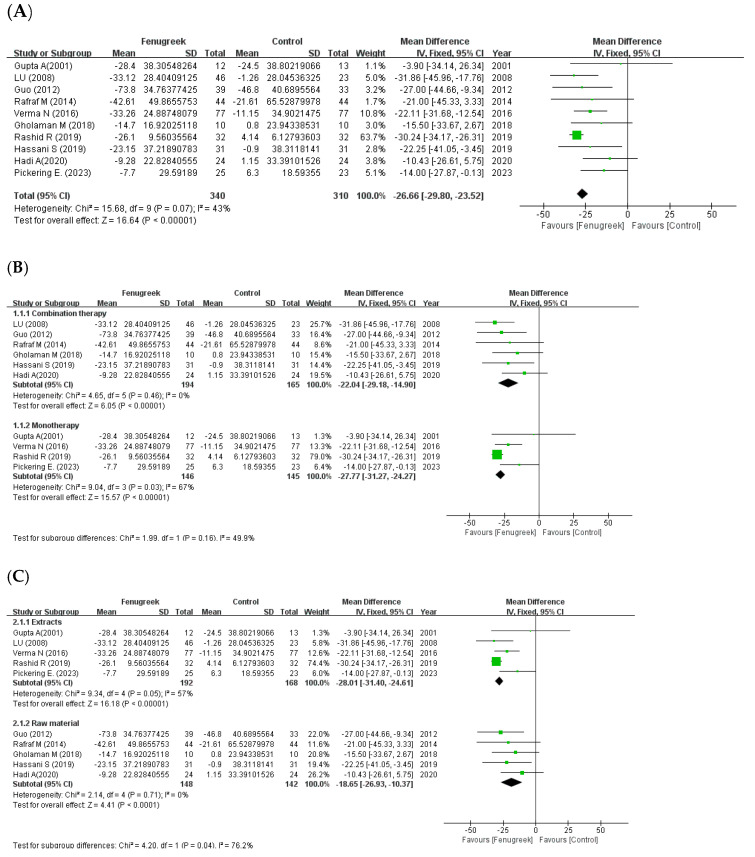
Forest plot for the effect of fenugreek on fasting blood glucose (FBG) (panel (**A**): all studies, panel (**B**): meta-analysis of combination therapy vs. monotherapy, panel (**C**): meta-analysis of extracts vs. raw material) [11,28,29,30,31,32,33,34,35,36]. 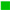 the position of the square represents the risk ratio, while the size of the square shows the weight of the study; 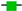 the black line indicates the 95% confidence interval of the result; 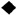 the diamond depicts overall pooled effect from the included studies.

A total of 10 studies were included in the meta-analysis of fasting blood glucose. FBG was significantly decreased in the intervention group compared to the control group (MD = −26.66; 95% CI: −29.80. −23.52; *p* < 0.00001; I^2^ = 43%; Figure 2A). In the subgroup analysis with treatment method (Figure 2B), there was significant a reduction in both combination therapy (MD = −22.04; 95% CI: −29.18, −14.90; *p* < 0.00001; I^2^ = 0%) and monotherapy (MD = −27.77; 95% CI: −31.27, −24.27; *p* < 0.00001; I^2^ = 67%). Furthermore, subgroup analysis based on intervention type (Figure 2C) also revealed significant differences in both groups. (extracts: MD = −28.01; 95% CI = −31.40, −54.61; *p* < 0.00001; I^2^ = 57%/raw material: MD = −18.65; 95% CI = −26.93, −10.37; *p* < 0.0001; I^2^ = 0%).

#### 3.3.2. 2 h Plasma Glucose during a 75 g Oral Glucose Tolerance Test

**Figure 3:**
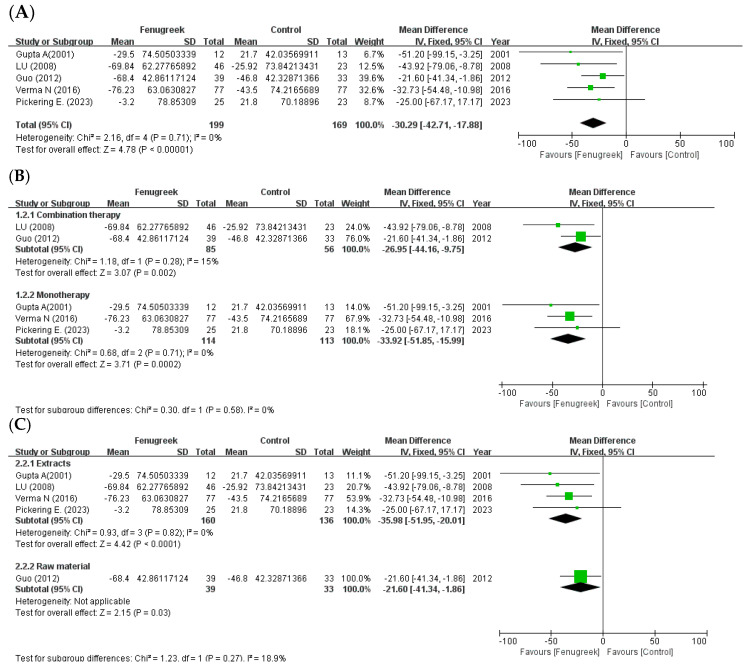
Forest plot for the effect of fenugreek on 2 h plasma glucose (2-hPG) (panel (**A**): all studies, panel (**B**): meta-analysis of combination therapy vs. monotherapy, panel (**C**): meta-analysis of extracts vs. raw material) [28,31,32,33,34]. 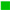 the position of the square represents the risk ratio, while the size of the square shows the weight of the study; 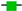 the black line indicates the 95% confidence interval of the result; 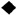 the diamond depicts overall pooled effect from the included studies.

In the meta-analysis of postprandial blood glucose, five studies showed significant improvement (MD = −30.29; 95% CI = −42.71, −17.88; *p* < 0.00001; I^2^ = 0%; Figure 3A). There were also significant differences in the treatment method (combination therapy: MD = −26.95; 95% CI = −44.16, −9.75; *p* = 0.002; I^2^ = 15%/monotherapy: MD = −33.92; 95% CI: −51.85, −15.99; *p* = 0.0002; I^2^ = 0%; Figure 3B) and intervention type (extracts: MD = −35.98; 95% CI: −51.95, −20.01; *p* < 0.0001; I^2^ = 0%/raw material: MD = −21.60; 95% CI = −41.34, −1.86; *p* = 0.03; Figure 3C).

#### 3.3.3. Homeostatic Model Assessment for Insulin Resistance 

**Figure 4:**
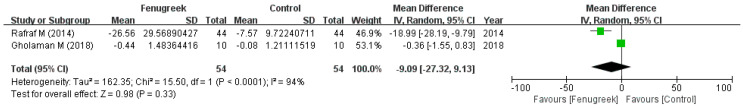
Forest plot for the effect of fenugreek on HOMA-IR [29,35]. 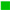 the position of the square represents the risk ratio, while the size of the square shows the weight of the study; 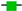 the black line indicates the 95% confidence interval of the result; 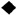 the diamond depicts overall pooled effect from the included studies.

Only two studies were included in the meta-analysis of HOMA-IR (Figure 4). Using a random effects model, the result showed that there was no significant decrease (MD = −9.09; 95% CI = −27.32, 9.13; *p* = 0.33; I^2^ = 94%). Subgroup analyses of the results were not presented because all these studies used raw material and were combination therapy.

#### 3.3.4. HbA1c

**Figure 5:**
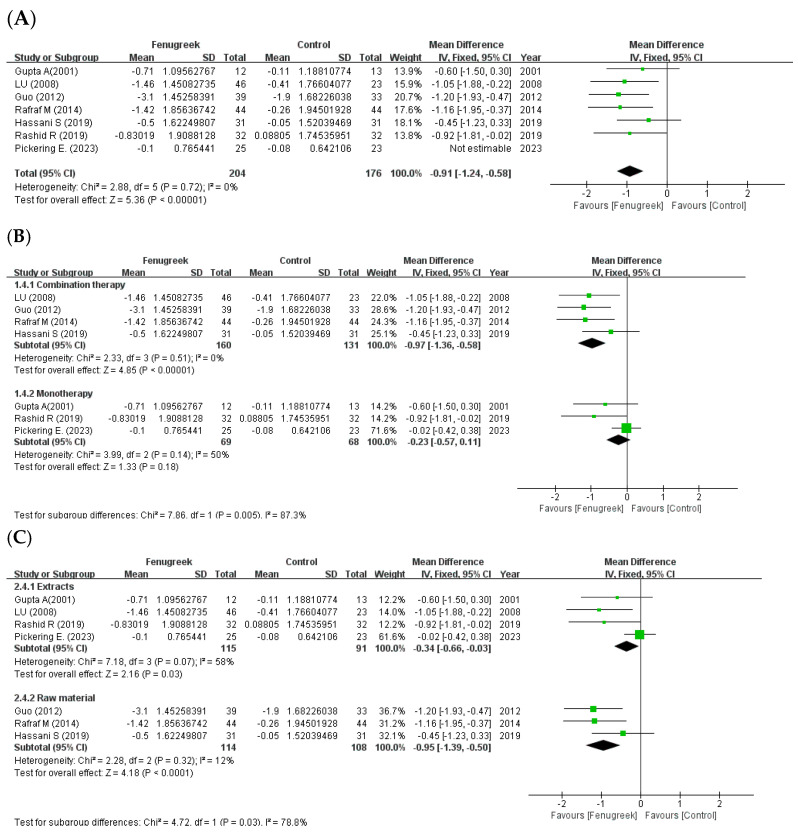
Forest plot for the effect of fenugreek on HbA1c (panel (**A**): all studies, panel (**B**): meta -analysis of combination therapy vs. monotherapy, panel (**C**): meta-analysis of extracts vs. raw material) [11,28,30,31,32,34,35]. 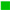 the position of the square represents the risk ratio, while the size of the square shows the weight of the study; 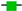 the black line indicates the 95% confidence interval of the result; 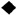 the diamond depicts overall pooled effect from the included studies.

Seven studies showed that there were significant differences in the meta-analysis of HbA1c (MD = −0.54; 95% CI = −0.80, −0.29; *p* < 0.0001; I^2^ = 58%; Figure 5A). In the subgroup analysis with treatment method (Figure 5B), combination therapy significantly improved HbA1c, while monotherapy did not show a significant improvement (combination therapy: MD = −0.97; 95% CI = −1.36, −0.58; *p* < 0.00001; I^2^ = 0%/monotherapy: MD = −0.23; 95% CI = −0.57, −0.11; *p* = 0.18; I^2^ = 50%). However, in the subgroup analysis with intervention type (Figure 5C), both extracts and raw material group revealed significant enhancement (extracts: MD = −0.34; 95% CI = −0.66, −0.03; *p* = 0.03; I^2^ = 58%/raw material: MD = −0.95; 95% CI = −1.39, −0.50; *p* < 0.0001; I^2^ = 12%).

### 3.4. Effect of Fenugreek Supplementation on Lipid Proflie and BMI

#### 3.4.1. Lipid Profile

**Figure 6:**
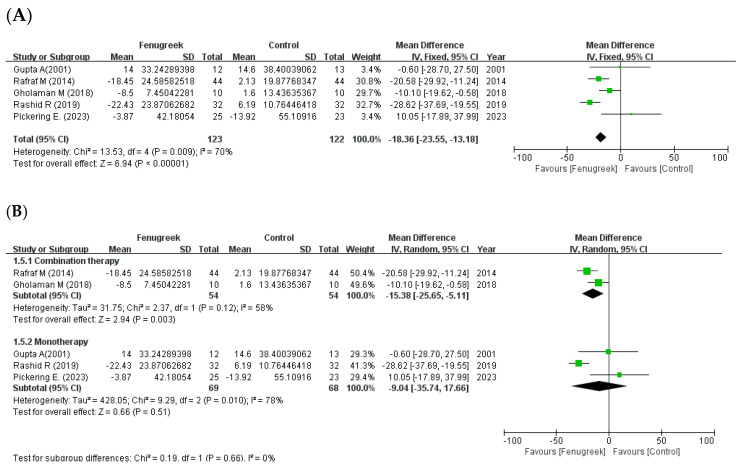

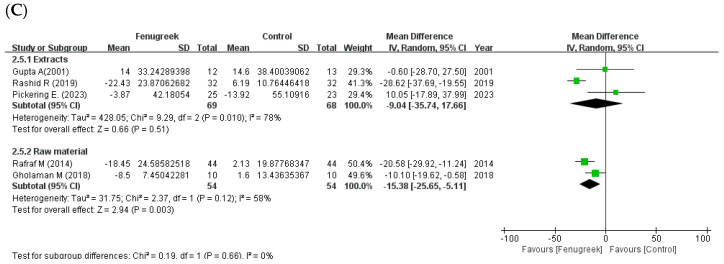
Forest plot for the effect of fenugreek on total cholesterol (TC) (panel (**A**): all studies, panel (**B**): meta-analysis of combination therapy vs. monotherapy, panel (**C**): meta-analysis of extracts vs. raw material) [11,28,29,31,35]. 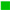 the position of the square represents the risk ratio, while the size of the square shows the weight of the study; 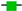 the black line indicates the 95% confidence interval of the result; 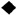 the diamond depicts overall pooled effect from the included studies.

**Figure 7:**
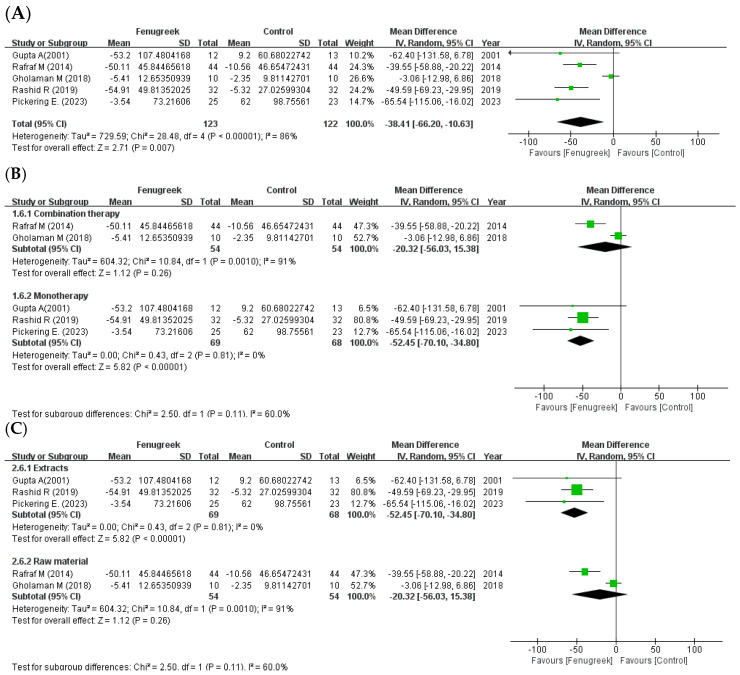
Forest plot for the effect of fenugreek on triglyceride (TG) (panel (**A**): all studies, panel (**B**): meta-analysis of combination therapy vs. monotherapy, panel (**C**): meta-analysis of extracts vs. raw material) [11,28,29,31,35]. 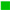 the position of the square represents the risk ratio, while the size of the square shows the weight of the study; 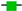 the black line indicates the 95% confidence interval of the result; 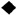 the diamond depicts overall pooled effect from the included studies.

**Figure 8:**
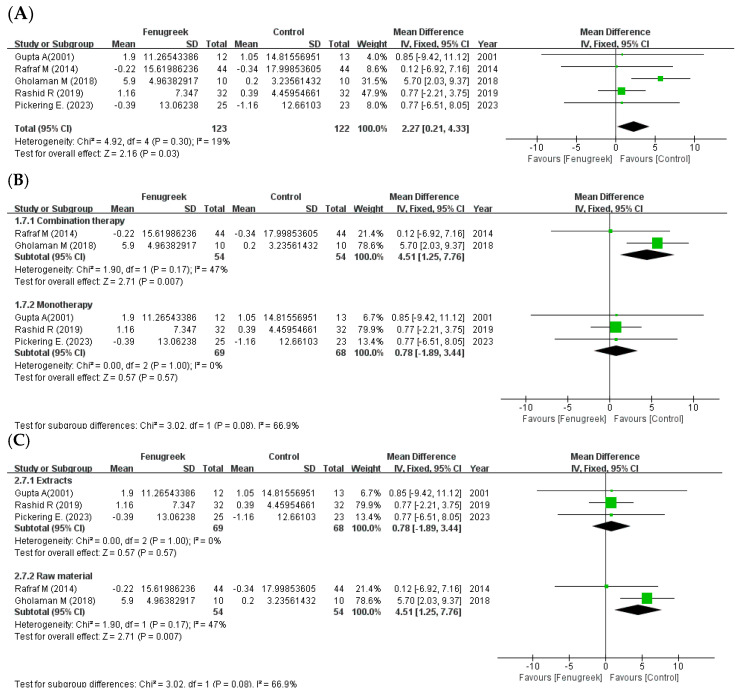
Forest plot for the effect of fenugreek on high density lipoprotein cholesterol (HDL-C) (panel (**A**): all studies, panel (**B**): meta-analysis of combination therapy vs. monotherapy, panel (**C**): meta-analysis of extracts vs. raw material) [11,28,29,31,35]. 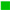 the position of the square represents the risk ratio, while the size of the square shows the weight of the study; 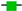 the black line indicates the 95% confidence interval of the result; 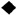 the diamond depicts overall pooled effect from the included studies.

**Figure 9:**
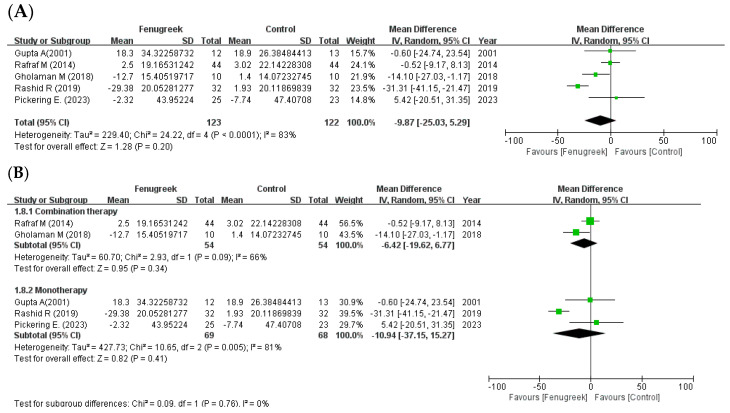

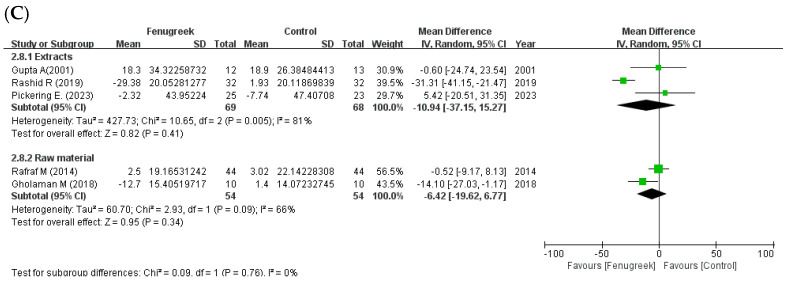
Forest plot for the effect of fenugreek on low density lipoprotein cholesterol (LDL-C) (panel (**A**): all studies, panel (**B**): meta-analysis of combination therapy vs. monotherapy, panel (**C**): meta-analysis of extracts vs. raw material) [11,28,29,31,35]. 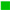 the position of the square represents the risk ratio, while the size of the square shows the weight of the study; 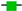 the black line indicates the 95% confidence interval of the result; 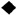 the diamond depicts overall pooled effect from the included studies.

Five studies reported that there were significant improvements in TC (MD = −18.36; 95% CI = −23.55, −13.18; *p* < 0.00001; I^2^ = 70%; Figure 6A), TG (MD = −38.41; 95% CI = −66.20, −10.63; *p* = 0.007; I^2^ = 86%; Figure 7A), and HDL-C (MD = 2.27; 95% CI = 0.21, 4.33; *p* = 0.03; I^2^ = 19%; Figure 8A). However, LDL-C was not reduced significantly (MD = −9.87; 95% CI = −25.03, 5.29; *p* = 0.20; I^2^ = 83%; Figure 9A). The random effects model was used in the meta-analysis of TG and LDL-C. In the subgroup analysis with treatment method (Figure 6B, Figure 7B, Figure 8B and Figure 9B), combination therapy significantly improved TC (MD = −15.38; 95% CI = −25.65, −5.11; *p* = 0.003; I^2^ = 58%) and HDL-C (MD = 4.51; 95% CI = 1.25, 7.76; *p* = 0.007; I^2^ = 47%), except for TG (MD = −20.32; 95% CI = −56.03, 15.38; *p* = 0.26; I^2^ = 91%) and LDL-C (MD = −6.42; 95% CI = −19.62, 6.77; *p* = 0.34; I^2^ = 66%). Monotherapy decreased TC (MD = −9.04; 95% CI = −35.74, 17.66; *p* = 0.51; I^2^ = 78%) and TG (MD = −52.45; 95% CI = −70.10, −34.80; *p* < 0.00001; I^2^ = 0%) significantly, while HDL-C (MD = 0.78; 95% CI = −1.89, 3.44; *p* = 0.57; I^2^ = 0%) and LDL-C (MD = −10.94; 95% CI = −37.15, 15.27; *p* = 0.41; I^2^ = 81%) were not changed significantly. The result of subgroup analysis with intervention type (Figure 6C, Figure 7C, Figure 8C and Figure 9C) revealed that TC (MD = −9.04; 95% CI = −35.74, 17.66; *p* = 0.51; I^2^ = 78%) and TG (MD = −52.45; 95% CI = −70.10, −34.80; *p* < 0.00001; I^2^ = 0%) were decreased significantly in extracts compared to HDL-C (MD = 0.78; 95% CI = −1.89, 3.44; *p* = 0.57; I^2^ = 0%) and LDL-C (MD = −10.94; 95% CI = −37.15, 15.27; *p* = 0.41; I^2^ = 81%). In raw material, TC (MD = −15.38; 95% CI = −25.65, −5.11; *p* = 0.003; I^2^ = 58%) and HDL-C (MD = 4.51; 95% CI = 1.25, 7.76; *p* = 0.007; I^2^ = 47%) were significant, whereas TG (MD = −20.32; 95% CI = −56.03, 15.38; *p* = 0.26; I^2^ = 91%) and LDL-C (MD = −6.42; 95% CI = −19.62, 6.77; *p* = 0.34; I^2^ = 66%) showed no significant differences in the same group.

#### 3.4.2. Body Mass Index (BMI)

**Figure 10:**
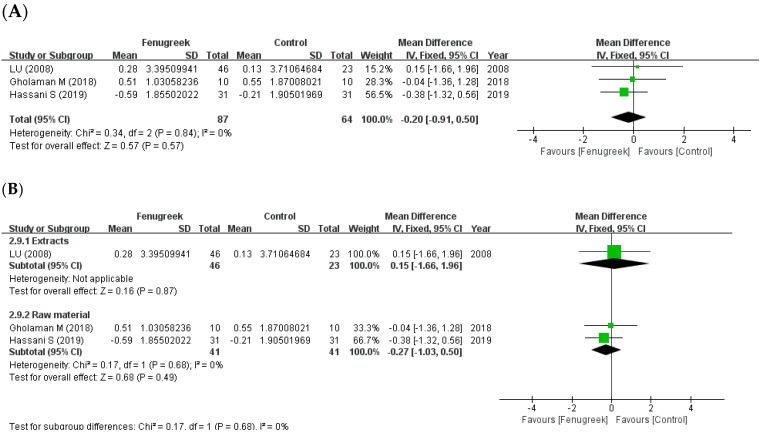
Forest plot for the effect of fenugreek on body mass index (BMI) (panel (**A**): all studies, panel (**B**): meta-analysis of extracts vs. raw material) [29,32,37]. 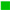 the position of the square represents the risk ratio, while the size of the square shows the weight of the study; 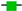 the black line indicates the 95% confidence interval of the result; 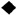 the diamond depicts overall pooled effect from the included studies.

The effect of fenugreek on BMI was reported in three studies (Figure 10A). The result showed that there was no significant decline in BMI (MD = −0.20; 95% CI = −0.91, 0.50; *p* = 0.57; I^2^ = 0%). All three studies were included in the combination group, so the result was equal to the overall effect. Also, the result of subgroup analysis based on intervention type (Figure 10B) showed that both extracts (MD = 0.15; 95% CI = −1.66, 1.96; *p* = 0.87) and raw material group (MD = −0.27; 95% CI = −1.03, 0.50; *p* = 0.49; I^2^ = 0%) have no significant effect.

### 3.5. Quality Assessment and Publication Bias 

**Figure 11:**
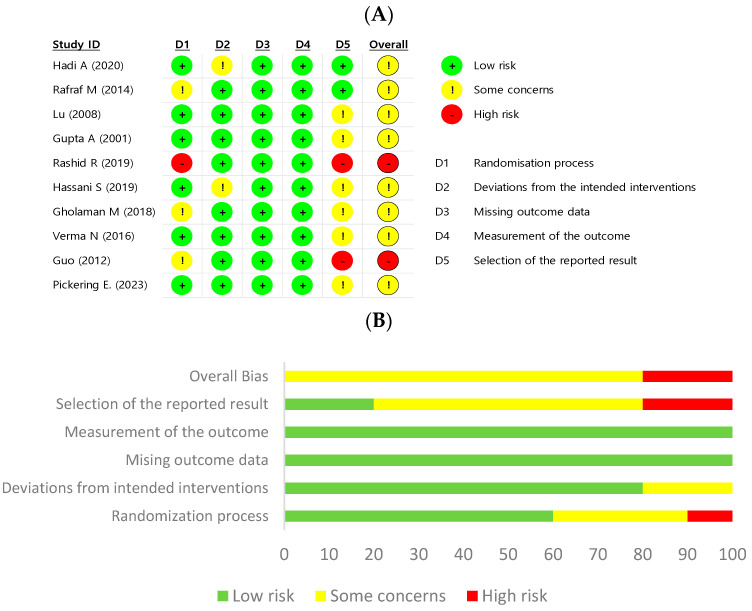
Quality assessment of included studies based on the Revised Cochrane risk of bias tool (ROB 2.0.). (**A**) A summary of the risk of bias in the included studies. (**B**) A graph of the risk in the bias of included studies [11,28,29,30,31,32,33,34,35,36].

**Figure 12:**
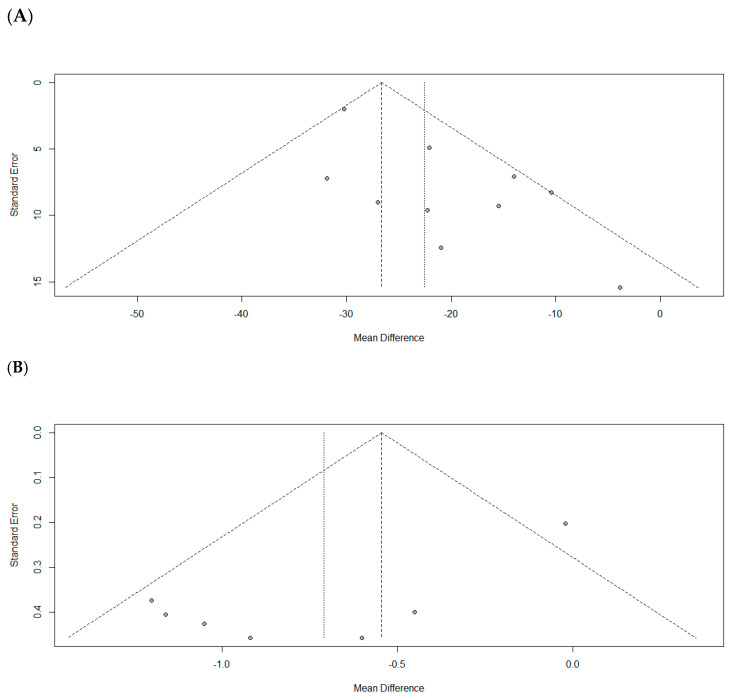
Funnel plot (**A**): fasting blood glucose (FBG) [11,28,29,30,31,32,33,34,35,36] (**B**): HbA1c [11,28,30,31,32,34,35].

Quality assessment by using the ROB 2.0. is described in Figure 11. Regarding the randomization process, most of the included studies [11,28,29,30,31,34,36] reported randomization, but they did not specify the method or allocation sequence. Two studies [30,36] were assessed for ‘some concerns’ in the domain of ‘deviations from intended interventions’ because they performed per-protocol (PP) analysis. Also, the overall quality of the domain ‘selection of reported results’ seemed to be poor. Because almost all studies did not mention whether the analysis was completed before outcome data were unblinded, it affected this poor quality. However, statisticians were blinded in two studies [35,36], so we regarded outcome data of these studies to be blinded until the analysis was accomplished. Some studies omitted part of the outcome data which they had planned they would measure, also leading to the poor quality of these studies [11,30,34]. The evaluation of publication bias based on funnel plot and Egger’s test indicated that there was no potential bias in each outcome, except for FBG and HbA1c (FBG: *p* = 0.0109/HbA1c: *p* = 0.0189; Figure 12). Thus, we speculated that most of results were representative of the actual effect.

### 3.6. Sensitivity Analysis

The heterogeneity of HOMA-IR, HbA1c, TC, TG, and LDL-C was more than 50%, so we had to conduct sensitivity analysis of them. However, despite the high heterogeneity of HOMA-IR, we could not conduct a sensitivity analysis because there were only two included studies. Thus, sensitivity analysis was performed on HbA1c, TC, TG, and LDL-C. In the case of HbA1c, the heterogeneity decreased with the omission of Guo et al., 2012 (from 58% to 53%), and Rafraf M et al., 2014 (from 58% to 57%), while the overall effect was affected but remained within a significant range. In Pickering E et al., 2023, the heterogeneity changed remarkably from 58% to 0% and the overall effect was improved. The exclusion of Rashid R et al., 2019, and Gholaman M et al., 2018, reduced the heterogeneity of TC and did not affect the overall effect. The heterogeneity of TC decreased from 68% to 41% in Rashid R et al., 2019, and from 68% to 52% in Gholaman M et al., 2018. Likewise, the removal of Rashid R et al., 2019, and Gholaman M et al., 2018, decreased the heterogeneity of TG, but it affected the overall effect. In Rashid R et al., 2019, heterogeneity was reduced from 88% to 85% and the overall effect changed to being non-significant (MD = −26.84; 95% CI = −60.12, 6.44; *p* = 0.11; I^2^ = 85%). In Gholaman M et al., 2018, the heterogeneity was reduced from 88% to 0% and the overall effect was statistically improved (MD = −45.17; 95% CI = −58.68, −31.66; *p* < 0.00001; I^2^ = 0%). In the case of LDL-C, the exclusion of Rafraf M et al., 2014, and Rashid R et al., 2019, decreased heterogeneity and affected the overall effect like TG. In Rafraf M et al., 2014, the heterogeneity reduced from 87% to 75% and the overall effect changed to be statistically significant (MD = −17.84; 95% CI = −34.42, −1.25; *p* = 0.04; I^2^ = 75%). In Rashid R et al., 2019, the heterogeneity reduced from 87% to 34%, and the overall effect change was worse (MD = −5.17; 95% CI = −14.64, 4.31; *p* = 0.29; I^2^ = 34%).

## 4. Discussion

### 4.1. Principal Findings

Our study found that fenugreek supplementation significantly improved all other glycemic control parameters (FBG, 2-hPG and HbA1c) and lipid profile (TC, TG and HDL-C), except for HOMA-IR, LDL-C, and BMI.

FBG, 2-hPG, and HbA1c are commonly used as representative blood glucose indicators and diagnostic criteria for diabetes by the American Diabetes Association (ADA) and the American Association of Clinical Endocrinologists (AACE). 

Bioactive compounds of fenugreek have a significant effect on glycemic control. According to Fuller S. et al., 2015 [38], diosgenin, a steroidal saponin, maintains insulin signaling and glucose homeostasis, and 4-OH-Ile stimulates insulin secretion. Also, the abundant dietary fiber of fenugreek, such as galactomannan, inhibits glucose and lipid absorption in the digestive system. These hypoglycemic effects were also confirmed in our study. 

Insulin resistance refers to impaired sensitivity to insulin mediated glucose disposal, resulting in a compensatory increase in beta-cell insulin production. It can lead to T2DM, metabolic syndrome and dyslipidemia. Although there is not a general clinical definition for insulin resistance, a number of clinically useful surrogate measures, such as HOMA-IR, are used to test insulin resistance [39,40]. 

In our study, two studies were included in the analysis of HOMA-IR. Out of the ten papers we selected, only two studies measured insulin resistance using HOMA-IR. Most studies were primarily designed to focus on the effect of fenugreek on the control of blood glucose, and as a result, HOMA-IR, an indicator of insulin resistance, was not measured. The heterogeneity of HOMA-IR was the highest among all results (94%), because one study [28] reported HOMA-IR values that were completely out of bounds (from 4.83 to 31.39). In other studies that measured HOMA-IR in diabetes and metabolic syndrome, mean values of HOMA-IR were 3.49 [41], 1.89 ± 1.37 [42], and 4.402 ± 2.794 [43]. Also, one study reported the median value as 3.39 (2.39–4.62) [44]. Therefore, we assumed that Rafraf M et al., 2014, has a numerical error compared to the findings of other articles. Furthermore, it is reported that the demographic characteristics including age, gender, and race influenced the cut-off values of HOMA-IR. So, we assumed that there are fewer studies that use HOMA-IR as an outcome compared to other outcomes [37,45].

In the insulin-resistant rodents model, fenugreek significantly reduced HOMA-IR in high-fructose-fed insulin-resistant rats [46]. However, in a clinical study for prediabetes, fenugreek did not show a significant effect on HOMA-IR compared to the placebo group during a 3 years follow-up [47]. These study results indicate that fenugreek does not have a clear effect on HOMA-IR with long-term administration. 

In our study, the consumption of fenugreek significantly improved TC and HDL levels, but there was no significant change observed in LDL levels.

Dyslipidemia is a lipid metabolism disorder characterized by the state of hypercholesterolemia, hypertriglyceridemia, elevated levels of LDL-C, and reduced levels of HDL-C. Insulin deficiency and insulin resistance in T2DM influence the enzymes and metabolic pathways involved in lipid metabolism [48]. The presence of small dense low-density lipoproteins (sdLDL), which are known to be more atherogenic than large LDL particles, is a typical characteristic of dyslipidemia caused by insulin resistance [49,50]. 

In Austin MA et al., 2014 [51], TG is associated with sdLDL, because TG-rich lipoproteins act as a major role in the formation of sdLDL particles. In fact, many studies have shown that sdLDL is present in the blood when TG levels exceed 132 mg/dL. HDL, on the other hand, plays a major role in reverse cholesterol transport, which is one of the protective effects of HDL against arteriosclerotic cardiovascular disease (ASCVD) [52]. 

The results of our study indicated that although fenugreek did not have a significant effect on LDL, it decreased TC and increased HDL. Notably, it significantly reduced TG levels, which are known to contribute to the formation of sdLDL in ASCVD. Based on these findings, we postulated that fenugreek may be useful for preventing the development of ASCVD in individuals with diabetic dyslipidemia.

BMI is currently used as an index that reflects an individual’s level of fatness based on anthropometric height and weight characteristics [53]. In obese individuals, there is an increase in the levels of substances involved in the development of insulin resistance, such as nonesterified fatty acids (NEFAs), glycerol, hormones, cytokines, and proinflammatory substances. Thus, obesity is considered a risk factor for diabetes, and it is necessary to control it for prevention and management of diabetes. 

Our study indicated that fenugreek supplementation did not have a significant effect on BMI. In the study by Hassani SS et al., 2019, it reported that BMI improved significantly after fenugreek intake in individuals with T2DM. However, several meta-analyses have shown that fenugreek could not significantly reduce BMI [48,54,55]. In the study by Kumar P et al., 2014, BMI was rather increased in high-fat-diet-induced obese rats after being fed the aqueous extract of fenugreek. In conclusion, fenugreek does not have an obvious effect on BMI. Therefore, it would be challenging to achieve improvement in diabetes mellitus with obesity by using fenugreek alone. Thus, additional therapy for obesity is needed for more effective management.

### 4.2. Subgroup Analysis 

In subgroup analysis, both FBG and 2-hPG showed significant reductions regardless of the treatment method and intervention type. FBG and 2-hPG indicated more reductions in the monotherapy and extracts groups. On the other hand, HbA1c showed improvement in both intervention type groups, but in the treatment method analysis, it exhibited a significant decline only in the combination therapy group. This result suggests that fenugreek can be considered a potential nutritional and dietary supplement for glycemic control in individuals with prediabetes. Moreover, fenugreek can be applied to support blood glucose control in individuals with diabetes who are concurrently receiving anti-diabetic medication. In raw material groups, fenugreek is classified into fenugreek seeds and fenugreek powder. The majority of individuals consume fenugreek in powder form, often mixed with yogurt or water. We found that the seed form exhibited a greater reduction in FBG levels compared to the powder form (MD = −21.42; −16.58) in Figure S1. Nevertheless, due to substantial variations in both the duration of administration and dosage, we cannot conclusively determine the superiority of the seed form over the powder form. 

In the extracts groups, we divided fenugreek extracts into two categories: those extracted using a solvent and those containing specific compounds of fenugreek. In the former category, two studies [28,31] reported the use of a hydroalcoholic extract, while other studies did not specify the solvent used for extraction. In the latter category, saponin and galactomannan of fenugreek were used as interventions. Our study revealed a significant reduction in FBG levels with galactomannan extracts compared to saponin extracts within the same duration (MD = −30.24; −25.19) in Figure S2. Thus, we assume that galactomannan extracts exhibit enhanced efficacy compared to saponin extracts. In conclusion, galactomannan extracts of fenugreek as a dietary supplement and medicinal intervention, appears to be a more reasonable choice compared to saponin extracts. 

Fenugreek contains many chemical compounds such as fibers [34], 4-OH-Ile, polyphenol, stilbenes, rhaponticin, and diosgenin [35], which demonstrated effects in controlling blood glucose and lipid levels. These compounds have unique mechanisms that contribute to their effects on blood glucose and lipid levels [33,34,35]. However, currently, there is no evidence on which compounds are more effective or how to extract them more effectively. Thus, further studies are needed to determine the most effective extraction method.

Regarding the lipid profile, TC and HDL-C significantly improved in the combination therapy and raw material groups, while TG levels were reduced in monotherapy and extracts. Therefore, our study revealed that the improvement of lipid profiles may be influenced by the treatment method and intervention type of fenugreek. However, it is important to note that the studies classified as combination therapy were the same as those in the raw material group, and the studies categorized as monotherapy were the same as those in the extracts group. This aspect should be considered when interpreting the results, and it cannot confirm which factor between treatment method and intervention type had more influence on result.

### 4.3. Sensitivity Analysis

We found that one study [28] had the largest effect on the heterogeneity of HbA1c in the treatment method; this study only targeted prediabetes and excluded T2DM. HbA1c reflects the average plasma glucose level over the past two to three months [56]. According to Chakarova N et al., 2019 [57], glucose variability is relatively large in prediabetes, so it may take a long time to improve HbA1c. Thus, fenugreek may not improve HbA1c in prediabetes. However, it has a positive effect on FBG and 2-hPG, indicating that it can be a potentially helpful option to manage prediabetes.

In the sensitivity analysis of BMI, two studies [29,35] using BMI as an outcome measurement had different inclusion criteria of BMI. This disparity in the baseline of anthropological characteristics of the participants is thought to be the cause of the heterogeneity. It is known that lifestyle modifications effectively improve FBG, WC, SBP, DBP, and TG, thereby preventing the progression of prediabetes to T2DM and alleviating symptoms associated with T2DM. Thus, we assume that differences in participant compliance with diet and exercise interventions contributed to the heterogeneity of results in FBG. 

### 4.4. Toxicity and Adverse Effect

Two studies [29,30] did not report any adverse effects, while Gholaman M et al., 2018 [29], concluded that fenugreek is safe. Except for these two studies, eight additional studies indicated the absence of liver or renal dysfunctions. Nevertheless, three studies [31,32,34] reported cases of abdominal distension, stomach discomfort, nausea, and diarrhea, and one study [28] reported light-headedness. However, these symptoms resolved without the need for special treatment. 

Verma MK et al., 2021 [13], discovered that fenugreek has a gastroprotective effect by reducing the volume of gastric juice and total acidity by antagonizing the H+/K+-ATPase pump. Proton Pump Inhibitors (PPIs), which act through a similar mechanism, have reported gastrointestinal disturbance during short-term administration, but they are generally well-tolerated by individuals. Likewise, the regulatory effect of fenugreek on gastric acid secretion may result in minor gastrointestinal side effects in some individuals, but these effects are tolerable. Additionally, apart from some gastrointestinal side effects, none of the included studies reported any serious adverse effects associated with fenugreek intake [58].

In Mowla A et al., 2009 [59], the results of a toxicity test on fenugreek suggested that fenugreek can be considered as an alternative for diabetic treatment with little to no side effects. Kandhare AD et al., 2019 [60] found that the standardized extract of fenugreek has a broad margin of safety based on seventeen toxicity studies. Therefore, despite the slight gastrointestinal side effects, fenugreek can be used as an alternative medicine without toxicity.

## 5. Strengths and Limitations

Our study has distinguished characteristics compared to other studies. Firstly, we included individuals with both T2DM and prediabetes. By managing these individuals, it may be possible to reduce the number of people who develop severe diabetes, and our study aimed to confirm the role of fenugreek in preventing diabetes.

Secondly, we investigated lipid profile as an outcome measurement, which is closely correlated with diabetic dyslipidemia [61]. By examining the lipid profile as an outcome, we intended to prove the efficacy of fenugreek in managing lipid profiles associated with diabetic complications.

Lastly, subgroup analyses were conducted based on treatment method (monotherapy vs. combination therapy) and intervention type (extracts vs. raw material). This approach enables us to assess the applicability of using fenugreek alone without the need for anti-diabetic drugs. Additionally, we could identify the most effective form of fenugreek and provide valuable data for the development of dietary supplements and alternative medicine using the active compounds of fenugreek. 

However, our study has several limitations. Firstly, the methods of fenugreek extraction, the preparation of intact part of fenugreek, dosage, and duration of the treatment were not standardized, so we speculated that these differences contributed to the variation in study results. Conducting additional RCTs and accumulating information on these factors would allow for subgroup analyses in diverse directions to determine the most effective and safe approach to fenugreek administration. Secondly, the number of included studies and participants in our analysis was insufficient to establish a significant effect of fenugreek on T2DM and prediabetes. Lastly, the overall quality of the included studies was considered poor based on the quality assessment. Therefore, larger-scale RCTs with high quality methodologies are necessary to confirm the role of fenugreek as a treatment for hyperglycemia. These further studies are expected to reveal the relationship between the active compounds of fenugreek and their impact on lowering blood glucose levels in the human body. Furthermore, they can contribute to establish standardized guidelines for the use of fenugreek in the prevention and treatment of diabetes.

## 6. Conclusions

This study revealed that fenugreek could improve overall glycemic control parameters and lipid profile. Especially, it significantly reduced FBG, 2-hPG, and HbA1c and improved TC, TG, and HDL-C. But there were no significant differences in HOMA-IR, LDL-C, and BMI. Adverse events were generally mild gastrointestinal side effects, with no reports of severe adverse events or hepatic or renal toxicity. These results suggest that fenugreek may be an effective and safe therapeutic option for individuals with T2DM. Further studies are needed to explore its long-term effects and potential benefits for other health outcomes.

## Figures and Tables

**Table 1:** Search terms.

Databases	Search Terms	
PubMed	(“Trigonella” [Mesh] OR “fenugreek seed meal” [Supplementary Concept]) AND “Glucose Metabolism Disorders” [Mesh]	
EMBASE	(‘diabetes mellitus’/exp/mj OR ‘dysglycemia’/exp/mj OR ‘glucose intolerance’/exp/mj OR ‘hyperglycemia’/exp/mj OR ‘insulin resistance’/exp/mj OR ‘glycosuria’/exp/mj OR ‘hyperinsulinism’/exp/mj) AND (‘trigonella’/exp/mj OR ‘fenugreek’/exp/mj OR ‘fenugreek extract’/exp/mj)	
Cochrane	#1	“Glucose Metabolism Disorder [MeSH]” AND “Trigonella [MeSH]”
	#2	(“fenugreek*” OR “fenugreek extract*” OR “fenugreek seed meal” OR “trigonella*”) AND (“hyperglycemia” OR “hyperinsulinism” OR “dysglycemia” OR “glucose intolerance” OR “diabet*” OR “insulin resistance” OR “glucose metabolism disorder*” OR “Glycosuria”)
	#3	#1 OR #2
CNKI	“葫芦巴” AND (“血糖” OR “糖尿”)	
KISS, RISS, Science ON	“호로파” OR “胡盧巴” OR “胡蘆巴” OR “葫蘆巴” OR “葫盧巴”	

**Table 2:** PICOS for inclusion criteria.

Parameter	Inclusion Criteria
Population	Human with high glucose level (FBG ≥ 100 mg/dL, HbA1c ≥ 5.7%)
Intervention	Fenugreek seed or its extracts
Comparisons	Placebo or no treatment
Outcomes	Fasting blood glucose (FBG), 2 h plasma glucose during a 75 g oral glucose tolerance test (OGTT) (2-hPG), homeostatic model assessment for insulin resistance (HOMA-IR), glycosylated hemoglobin (HbA1c)/total cholesterol (TC), triglyceride (TG), high density lipoprotein (HDL-C), low density lipoprotein (LDL-C), body mass index (BMI)
Study design	RCTs in parallel design

**Table 3:** PICOS for Exclusion criteria.

Parameter	Exclusion Criteria
Population	Healthy people (FBG < 100 mg/dL, HbA1c < 5.7%)
Intervention	Combination of fenugreek with other herbs or non-seed part of fenugreek
Comparisons	Comparison of the hyperglycemic drugs
Study design	Non-RCTs (cohort studies, RCTs in cross-over design), acute studies ≤ 10 days

**Table 4:** Characteristics of included studies.

References	Study Design	Duration	Participants Age (Mean or Range) Male: Female Medication	Intervention Group		Control Group		Additional Therapy	Primary Outcomes	Adverse Events (Group, N)
				Size, N	Intervention [Type, Daily Dose]	Size, N	Control			
Arvind Gupta et al. [31]	DB, PL	2 mts	Mild to moderate T2DM (FBG < 200 mg/dL) I: 49.16 years/C: 52.83 yrs 19:6 NR	12	Fenugreek [HS, 1 gm, 2 capsule × 2]	13	Usual care (diet control, exercise) + placebo (2 capsules × 2)	SU (I: 3, C:1) SU + BI (I: 7, C: 9)	− Intervention group (a) ↓ FBG, 2-hPG, HbA1c, TG (b) ↑ HDL No improvements: (c) ↑ TC, LDL − Control group (a) ↓ FBG, HbA1c (b) ↑ HDL (c) No improvements: ↑ 2-hPG, TC, TG, LDL	dyspepsia and mild abdominal distension (I, 5)
Lu Fu-rong et al. [32]	DB, PL	12 wks	T2DM poorly controlled by SU (7 mmol/L < FBG < 13 mmol/L, ±1.5 mmol/L) 25–65 years 38:31 SU	46	Fenugreek extract [TFGs capsules (0.35 g/pill), 6 capsules × 3] + SU	23	Chinese yam placebo (0.35 g/pill, 6 capsules × 3) + SU	None	− Intervention group (a) ↓ FBG, 2-hPG, HbA1c (b) No improvements: ↑ BMI − Control group (a) ↓ FBG, 2-hPG, HbA1c (b) No improvements: ↑ BMI	stomach discomforts and nausea (I.2)/diarrhea (I,1)
Guo et al. [34]	PL	16 wks	T2DM (FBG ≥ 7 mmol/L, 2 hPG ≥ 11.1 mmol/L) with diabetic nephropathy I: 58 years/C:59 years 39:33 NR	39	Fenugreek [FS, 10 g × 3] + VAL (80 mg once a day) + diet, exercise	33	VAL (80 mg once a day) + diet, exercise	None	− Intervention group (a) ↓ FBG, 2-hPG, HbA1c − Control group (b) ↓ FBG, 2-hPG, HbA1c	abdominal distension and stomach discomforts (I, 3)/abdominal distension (C, 2)
Maryam Rafraf et al. [35]	TB, PL	8 wks	T2DM (diabetic history ≥ 6 months, FBG ≥ 126 mg/dL) with BMI ≤ 35 kg/m^2^ 30–60 years 10:78 MET/GLI	44	Fenugreek [PS, 5 g × 2] + MET/GLI + usual diet	44	Placebo (wheat starch, 2.5 g × 2) + MET/GLI + usual diet	None	− Intervention group (a) ↓ FBG, HOMA-IR, HbA1c, TC, TG (b) No improvements: ↓ HDL ↑ LDL − Control group (a) ↓ FBG, HOMA-IR, HbA1c, TG (b) No improvements: ↑ TC, LDL ↓ HDL	None
Narsingh Verma et al. [33]	DB, PL	90 days	T2DM (diabetic history < 5 years, FBG < 180 mg/dL, HbA1c > 7.5%) 25–60 years 108:46 MET/SU	77	Fenfuro [500 mg capsule, 2 capsules] + MET + diet (< 2000 kcal/day, protein 18~22%, carbohydrate 52~56%, fat 22~26%)	77	Placebo (di calcium phosphate, 2 capsules) + MET + diet (< 2000 kcal/day, protein 18~22%, carbohydrate 52~56%, fat 22~26%)	None	− Intervention group (a) ↓ FBG, PPBG, HbA1c − Control group (a) ↓ FBG, PPBG, HbA1c	None
Gholaman M et al. [29]	DB, PL	8 wks	Obese woman T2DM with BMI > 30 kg/m^2^ 44.2 years 0:40 Changed antidiabetic drugs	10	Fenugreek [FSs, 5 g + 100 g yogurt × 3] + antidiabetic drugs	10	Placebo (flavors) + 100 g yogurt + antidiabetic drugs	None	− Intervention group (a) ↓ FBG, HOMA-IR, TC, TG, LDL (b) ↑ HDL (c) No improvements: ↑ BMI − Control group (A) placebo (a) ↑ FBG (b) ↓ HOMA-IR, TG (c) No improvements: ↑ TC, LDL, BMI ↓ HDL (B) exercise (a) ↓ FBG, HOMA-IR, TC, TG, LDL, BMI (b) ↑ HDL (C) FSs + exercise (a) ↓ FBG, HOMA-IR, TC, TG, LDL, BMI (b) ↑ HDL	NR
						10	Aerobic exercise (running and walking) + antidiabetic drugs			
						10	FSs + exercise + antidiabetic drugs			
Raheela Rashid et al. [11]	SB, PL	12 wks	Newly diagnosed T2DM 30–60 years 29:35 NR	32	Fenugreek extract [galactomannan capsule, 1 g] + modified diabetic diet and exercise (walking)	32	Placebo (1 g) + modified diabetic diet and exercise (walking)	None	− Intervention group (a) ↓ FBG, HbA1c, TC, TG, LDL (b) ↑ HDL − Control group (a) ↓ FBG, HbA1c, TG, LDL (b) ↑ HDL (c) No improvements: ↑ TC	None
Seyyedeh Seddigheh Hassani et al. [30]	DB, PL	2 mts	T2DM(diabetic history ≥ 6 months, not insulin injection) with BMI < 35 kg/m^2^ 35–70 years 54:71 glycemic drugs	31	Fenugreek [PS, 5 g×2] + classic treatments	31	Placebo (wheat flour, 5 g × 2)	None	− Intervention group (a) ↓ FBG, HbA1C, BMI − Control group (A) Placebo (a) ↓ FBG, HbA1c, BMI (B) PS + nutrition training (a) ↓ FBG, HbA1c, BMI (C) placebo + nutrition training (a) ↓ FBG, HbA1c, BMI	NR
						31	PS (5 g × 2) + nutrition training (2 h)			
						32	Placebo (5 g × 2) + nutrition training (2 h)			
Amir Hadi et al. [36]	PL	8 wks	T2DM (diabetic history < 10 years) with BMI < 35 kg/m^2^ 30–65 years 22:26 antidiabetic drugs	24	Fenugreek with water [PS, 5 g × 3] + antidiabetic drug + nutritional recommendations	24	Nutritional recommendations	None	− Intervention group (a) ↑ FBG − Control group (a) ↑ FBG	None
Emily Pickering et al. [28]	DB, PL	12 wks	Prediabetes (FBG > 5.5 mmol/L, 2 hPG > 7.8 mmol/L) I: 58.2 years/C: 59.7 years 28:20 None	25	fenugreek [HS, 250 mg/tablet, 1 tablet × 2]	23	Placebo (maltodextrin, 1 tablets × 2)	None	− Intervention group (a) ↓ FBG, 2 hPG, HbA1C, TC, LDL, TG (b) ↓ HDL − Control group (a) ↓ HbA1C, TC, LDL (b) ↓ HDL (c) ↑ FBG, 2 hPG, TG	Light-headed (NR)

T2DM: Type 2 diabetes mellitus/SB: single-blinded/DB: double-blinded/TB: triple-blinded/OL: open label/PL: parallel/NR: not reported/PS: powdered fenugreek seeds/HS: hydro alcoholic extract of fenugreek seeds/FS: fenugreek seeds/TFGs: *Trigonella foenum-graecum* L. total saponins/FSs: Fenugreek seed supplement/I: intervention group/C: control group/SU: sulfonylurea/BI: Biguanides/VAL: valsartan/MET: metformin/GLI: glibenclamide/FBG: fasting blood glucose/2-hPG: 2 h plasma glucose during a 75 g oral glucose tolerance test (OGTT)/PPBG: post prandial blood glucose/HOMA-IR: homeostatic model assessment for insulin resistance/BMI: Body mass index/HbAlc: glycosylated hemoglobin/TC: total cholesterol/TG: triglyceride/HDL: high-density lipoprotein/LDL: low-density lipoprotein/NS: not significant.

## Data Availability

Not applicable.

## Supplementary Materials

The following supporting information can be downloaded at: https://www.mdpi.com/article/10.3390/ijms241813999/s1.

## References

[1] Diabetes Now Affects One in 10 Adults Worldwide. https://idf.org/news/diabetes-now-affects-one-in-10-adults-worldwide/.

[2] AlKurd R., Hanash N., Khalid N., Abdelrahim D.N., Khan M.A.B., Mahrous L., Radwan H., Naja F., Madkour M., Obaideen K. (2022). Effect of Camel Milk on Glucose Homeostasis in Patients with Diabetes: A Systematic Review and Meta-Analysis of Randomized Controlled Trials. Nutrients.

[3] Yang C.P., Lin C.C., Li C.I., Liu C.S., Lin W.Y., Hwang K.L., Yang S.Y., Chen H.J., Li T.C. (2015). Cardiovascular Risk Factors Increase the Risks of Diabetic Peripheral Neuropathy in Patients With Type 2 Diabetes Mellitus: The Taiwan Diabetes Study. Medicine.

[4] Jaiswal M., Schinske A., Pop-Busui R. (2014). Lipids and lipid management in diabetes. Best Pract. Res. Clin. Endocrinol. Metab..

[5] Gray N., Picone G., Sloan F., Yashkin A. (2015). Relation between BMI and diabetes mellitus and its complications among US older adults. South. Med. J..

[6] Leiherer A., Mundlein A., Drexel H. (2013). Phytochemicals and their impact on adipose tissue inflammation and diabetes. Vasc. Pharmacol..

[7] Radwan H., Hasan H., Hamadeh R., Hashim M., AbdulWahid Z., Hassanzadeh Gerashi M., Al Hilali M., Naja F. (2020). Complementary and alternative medicine use among patients with type 2 diabetes living in the United Arab Emirates. BMC Complement. Med. Ther..

[8] Chang H.-Y., Wallis M., Tiralongo E. (2007). Use of complementary and alternative medicine among people living with diabetes: Literature review. J. Adv. Nurs..

[9] Birdee G.S., Yeh G. (2010). Complementary and Alternative Medicine Therapies for Diabetes: A Clinical Review. Clin. Diabetes.

[10] Yeh G.Y., Eisenberg D.M., Kaptchuk T.J., Phillips R.S. (2003). Systematic review of herbs and dietary supplements for glycemic control in diabetes. Diabetes Care.

[11] Rashid R., Ahmad H., Ahmed Z., Rashid F., Khalid N. (2019). Clinical investigation to modulate the effect of fenugreek polysaccharides on type-2 diabetes. Bioact. Carbohydr. Diet. Fibre.

[12] Singh A.B., Tamarkar A.K., Shweta, Narender T., Srivastava A.K. (2010). Antihyperglycaemic effect of an unusual amino acid (4-hydroxyisoleucine) in C57BL/KsJ-db/db mice. Nat. Prod. Res..

[13] Kumar M., Verma M.K., Ranjan R., Kumar N., Ramanarayanan S. (2021). Bioactive effects and safety profiles of fenugreek (*Trigonella foenum-graecum* L.) for pharmaceutical and medicinal applications. Pharma Innov..

[14] Wang E., Wylie-Rosett J. (2008). Review of selected Chinese herbal medicines in the treatment of type 2 diabetes. Diabetes Educ..

[15] Saxena A., Vikram N.K. (2004). Role of selected Indian plants in management of type 2 diabetes: A review. J. Altern. Complement. Med..

[16] John L.J., Shantakumari N. (2015). Herbal Medicines Use During Pregnancy: A Review from the Middle East. Oman Med. J..

[17] Taylor W.G., Zulyniak H.J., Richards K.W., Acharya S.N., Bittman S., Elder J.L. (2002). Variation in diosgenin levels among 10 accessions of fenugreek seeds produced in western Canada. J. Agric. Food Chem..

[18] Demmers A., Korthout H., van Etten-Jamaludin F.S., Kortekaas F., Maaskant J.M. (2017). Effects of medicinal food plants on impaired glucose tolerance: A systematic review of randomized controlled trials. Diabetes Res. Clin. Pract..

[19] Heshmat-Ghahdarijani K., Mashayekhiasl N., Amerizadeh A., Teimouri Jervekani Z., Sadeghi M. (2020). Effect of fenugreek consumption on serum lipid profile: A systematic review and meta-analysis. Phytother. Res..

[20] Gong J., Fang K., Dong H., Wang D., Hu M., Lu F. (2016). Effect of fenugreek on hyperglycaemia and hyperlipidemia in diabetes and prediabetes: A meta-analysis. J. Ethnopharmacol..

[21] Page M.J., McKenzie J.E., Bossuyt P.M., Boutron I., Hoffmann T.C., Mulrow C.D., Shamseer L., Tetzlaff J.M., Akl E.A., Brennan S.E. (2021). The PRISMA 2020 statement: An updated guideline for reporting systematic reviews. BMJ.

[22] (2020). KFDA Notification No. 2020-73.

[23] Commission of Chinese Pharmacopoeia (2015). Pharmacopoeia of the People's Republic of China.

[24] Understanding A1C. https://diabetes.org/diabetes/a1c/diagnosis.

[25] Follmann D., Elliott P., Suh I., Cutler J. (1992). Variance imputation for overviews of clinical trials with continuous response. J. Clin. Epidemiol..

[26] Higgins J.P.T., Thomas J., Chandler J., Cumpston M., Li T., Page M.J., Welch V.A. Cochrane Handbook for Systematic Reviews of Interventions Version 6.3. www.training.cochrane.org/handbook.

[27] Bowden J., Tierney J.F., Copas A.J., Burdett S. (2011). Quantifying, displaying and accounting for heterogeneity in the meta-analysis of RCTs using standard and generalised Qstatistics. BMC Med. Res. Methodol..

[28] Pickering E., Steels E., Rao A., Steadman K.J. (2022). An Exploratory Study of the Safety and Efficacy of a *Trigonella foenum-graecum* Seed Extract in Early Glucose Dysregulation: A Double-Blind Randomized Placebo-Controlled Trial. Pharmaceutics.

[29] Gholaman M., Gholami M. (2018). Effect of Eight Weeks’ Endurance Training along with Fenugreek Ingestion on Lipid Profile, Body Composition, Insulin Resistance and VO2max in Obese Women’s with Type2 Diabetes. J. Med. Plants.

[30] Hassani S.S., Fallahi A., Esmaeili S.S., Gholami Fesharaki M. (2019). The Effect of Combined Therapy with Fenugreek and Nutrition Training Based on Iranian Traditional Medicine on FBS, HgA1c, BMI, and Waist Circumference in Type 2 Diabetic Patients: A Randomized Double Blinded Clinical Trial. J. Adv. Med. Biomed. Res..

[31] Gupta A., Gupta R., Lal B. (2001). Effect of *Trigonella foenum-graecum* (Fenugreek) Seeds on Glycaemic Control and Insulin Resistance in Type 2 Diabetes Mellitus: A Double Blind Placebo Controlled Study. J. Assoc. Physicians India.

[32] Lu F.R., Shen L., Qin Y., Gao L., Li H., Dai Y. (2008). Clinical observation on *Trigonella foenum-graecum* L. total saponins in combination with sulfonylureas in the treatment of type 2 diabetes mellitus. Chin. J. Integr. Med..

[33] Verma N., Usman K., Patel N., Jain A., Dhakre S., Swaroop A., Bagchi M., Kumar P., Preuss H.G., Bagchi D. (2016). A multicenter clinical study to determine the efficacy of a novel fenugreek seed (*Trigonella foenum-graecum*) extract (Fenfuro) in patients with type 2 diabetes. Food Nutr. Res..

[34] Guo Cheng-kun Z.Q. (2012). *Trigonella foenum graecum* combined with valsartan in treatment of diabetic nephropathy. Chin. J. Difficult Complicat. Cases.

[35] Rafraf M., Malekiyan M., Asghari-Jafarabadi M., Aliasgarzadeh A. (2014). Effect of Fenugreek Seeds on Serum Metabolic Factors and Adiponectin Levels in Type 2 Diabetic Patients. Int. J. Vitam. Nutr. Res..

[36] Hadi A., Arab A., Hajianfar H., Talaei B., Miraghajani M., Babajafari S., Marx W., Tavakoly R. (2020). The effect of fenugreek seed supplementation on serum irisin levels, blood pressure, and liver and kidney function in patients with type 2 diabetes mellitus: A parallel randomized clinical trial. Complement. Ther. Med..

[37] Khalili D., Khayamzadeh M., Kohansal K., Ahanchi N.S., Hasheminia M., Hadaegh F., Tohidi M., Azizi F., Habibi-Moeini A.S. (2023). Are HOMA-IR and HOMA-B good predictors for diabetes and pre-diabetes subtypes?. BMC Endocr. Disord..

[38] Fuller S., Stephens J.M. (2015). Diosgenin, 4-hydroxyisoleucine, and fiber from fenugreek: Mechanisms of actions and potential effects on metabolic syndrome. Adv. Nutr..

[39] Wilcox G. (2005). Insulin and Insulin Resistance. Clin. Biochem. Rev..

[40] Gayoso-Diz P., Otero-Gonzalex A., Rodriguez-Alvarez M.X., Gude F., Cadarso-Suarez C., García F., De Francisco A. (2011). Insulin resistance index (HOMA-IR) levels in a general adult population: Curves percentile by gender and age. The EPIRCE study. Diabetes Res. Clin. Pract..

[41] Horakova D., Stepanek L., Janout V., Janoutova J., Pastucha D., Kollarova H., Petrakova A., Stepanek L., Husar R., Martinik K. (2019). Optimal Homeostasis Model Assessment of Insulin Resistance (HOMA-IR) Cut-Offs: A Cross-Sectional Study in the Czech Population. Medicina.

[42] Yokoyama H., Emoto M., Fujiwara S., Motoyama K., Morioka T., Komatsu M., Tahara H., Koyama H., Shoji T., Inaba M. (2004). Quantitative insulin sensitivity check index and the reciprocal index of homeostasis model assessment are useful indexes of insulin resistance in type 2 diabetic patients with wide range of fasting plasma glucose. J. Clin. Endocrinol. Metab..

[43] Vladu I.M., Fortofoiu M., Clenciu D., Fortofoiu M.C., Padureanu R., Radu L., Cojan S.T.T., Radulescu P.M., Padureanu V. (2022). Insulin resistance quantified by the value of HOMA-IR and cardiovascular risk in patients with type 2 diabetes. Exp. Ther. Med..

[44] Diniz M.D.F.H.S., Beleigoli A.M.R., Schmidt M.I., Duncan B.B., Ribeiro A.L.P., Vidigal P.G., Benseñor I.M., Lotufo P.A., Santos I.S., Griep R.H. (2020). Homeostasis model assessment of insulin resistance (HOMA-IR) and metabolic syndrome at baseline of a multicentric Brazilian cohort: ELSA-Brasil study. Cad. Saúde Pública.

[45] Tahapary D.L., Pratisthita L.B., Fitri N.A., Marcella C., Wafa S., Kurniawan F., Rizka A., Tarigan T.J.E., Harbuwono D.S., Purnamasari D. (2022). Challenges in the diagnosis of insulin resistance: Focusing on the role of HOMA-IR and Tryglyceride/glucose index. Diabetes Metab. Syndr..

[46] Mohammadi A., Gholamhosseinian A., Fallah H. (2016). *Trigonella foenum-graecum* water extract improves insulin sensitivity and stimulates PPAR and gamma gene expression in high fructose-fed insulin-resistant rats. Adv. Biomed. Res..

[47] Gaddam A., Galla C., Thummisetti S., Marikanty R.K., Palanisamy U.D., Rao P.V. (2015). Role of Fenugreek in the prevention of type 2 diabetes mellitus in prediabetes. J. Diabetes Metab. Disord..

[48] Geberemeskel G.A., Debebe Y.G., Nguse N.A. (2019). Antidiabetic Effect of Fenugreek Seed Powder Solution (*Trigonella foenum-graecum* L.) on Hyperlipidemia in Diabetic Patients. J. Diabetes Res..

[49] Jialal I., Singh G. (2019). Management of diabetic dyslipidemia: An update. World J. Diabetes.

[50] Ormazabal V., Nair S., Elfeky O., Aguayo C., Salomon C., Zuniga F.A. (2018). Association between insulin resistance and the development of cardiovascular disease. Cardiovasc. Diabetol..

[51] Austin M.A., King M.C., Vranizan K.M., Krauss R.M. (1990). Atherogenic lipoprotein phenotype. A proposed genetic marker for coronary heart disease risk. Circulation.

[52] Khera A.V., Cuchel M., de la Llera-Moya M., Rodrigues A., Burke M.F., Jafri K., French B.C., Phillips J.A., Mucksavage M.L., Wilensky R.L. (2011). Cholesterol efflux capacity, high-density lipoprotein function, and atherosclerosis. N. Engl. J. Med..

[53] Nuttall F.Q. (2015). Body Mass Index: Obesity, BMI, and Health: A Critical Review. Nutr. Today.

[54] Roberts K.T. (2011). The potential of fenugreek (*Trigonella foenum-graecum*) as a functional food and nutraceutical and its effects on glycemia and lipidemia. J. Med. Food.

[55] Khodamoradi K., Khosropanah M.H., Ayati Z., Chang D., Nasli-Esfahani E., Ayati M.H., Namazi N. (2020). The Effects of Fenugreek on Cardiometabolic Risk Factors in Adults: A Systematic Review and Meta-analysis. Complement. Ther. Med..

[56] Nathan D.M., Turgeon H., Regan S. (2007). Relationship between glycated haemoglobin levels and mean glucose levels over time. Diabetologia.

[57] Chakarova N., Dimova R., Grozeva G., Tankova T. (2019). Assessment of glucose variability in subjects with prediabetes. Diabetes Res. Clin. Pract..

[58] Yibirin M., De Oliveira D., Valera R., Plitt A.E., Lutgen S. (2021). Adverse Effects Associated with Proton Pump Inhibitor Use. Cureus.

[59] Mowla A., Alauddin M., Rahman M.A., Ahmed K. (2009). Antihyperglycemic Effect of *Trigonella foenum-graecum* (Fenugreek) Seed Extract in Alloxan-Induced Diabetic Rats and Its Use in Diabetes Mellitus: A Brief Qualitative Phytochemical and Acute Toxicity Test on the Extract. Afr. J. Tradit. Complement. Altern. Med..

[60] Kandhare A.D., Thakurdesai P.A., Wangikar P., Bodhankar S.L. (2019). A systematic literature review of fenugreek seed toxicity by using ToxRTool: Evidence from preclinical and clinical studies. Heliyon.

[61] Verges B. (2015). Pathophysiology of diabetic dyslipidaemia: Where are we?. Diabetologia.

