# Supplementary material for: The Effect of Fenugreek in Type 2 Diabetes and Prediabetes: A Systematic Review and Meta-Analysis of Randomized Controlled Trials
Source: Int J Mol Sci. 2023 Sep 12;24(18):13999. doi: 10.3390/ijms241813999 (PMC10531284; doi:10.3390/ijms241813999)
Supplement: Supplementary file 1 [file ijms-24-13999-s001.zip › ijms-2590269-supplementary.pdf]

| Study or Subgroup | Seed Mean | Seed SD | Seed Total | Powder Mean | Powder SD | Powder Total | Weight | Mean Difference IV, Fixed, 95% CI | Year |
|---|---|---|---|---|---|---|---|---|---|
| **4.1.1 seed** | | | | | | | | | |
| Guo (2012) | -73.8 | 34.76377425 | 39 | -46.8 | 40.6895564 | 33 | 22.0% | -27.00 [-44.66, -9.34] | 2012 |
| Gholaman M (2018) | -14.7 | 16.92025118 | 10 | 0.8 | 23.94338531 | 10 | 20.8% | -15.50 [-33.67, 2.67] | 2018 |
| **Subtotal (95% CI)** | | | **49** | | | **43** | **42.8%** | **-21.42 [-34.08, -8.75]** | |
| Heterogeneity: Chi² = 0.79, df = 1 (P = 0.37); I² = 0% | | | | | | | | | |
| Test for overall effect: Z = 3.31 (P = 0.0009) | | | | | | | | | |
| **4.1.2 powder** | | | | | | | | | |
| Rafraf M (2014) | -42.61 | 49.8655753 | 44 | -21.61 | 65.52879978 | 44 | 11.6% | -21.00 [-45.33, 3.33] | 2014 |
| Hassani S (2019) | -23.15 | 37.21890783 | 31 | -0.9 | 38.3118141 | 31 | 19.4% | -22.25 [-41.05, -3.45] | 2019 |
| Hadi A(2020) | -9.28 | 22.82840555 | 24 | 1.15 | 33.39101526 | 24 | 26.2% | -10.43 [-26.61, 5.75] | 2020 |
| **Subtotal (95% CI)** | | | **99** | | | **99** | **57.2%** | **-16.58 [-27.53, -5.63]** | |
| Heterogeneity: Chi² = 1.03, df = 2 (P = 0.60); I² = 0% | | | | | | | | | |
| Test for overall effect: Z = 2.97 (P = 0.003) | | | | | | | | | |
| **Total (95% CI)** | | | **148** | | | **142** | **100.0%** | **-18.65 [-26.93, -10.37]** | |
| Heterogeneity: Chi² = 2.14, df = 4 (P = 0.71); I² = 0% | | | | | | | | | |
| Test for overall effect: Z = 4.41 (P < 0.0001) | | | | | | | | | |
| Test for subgroup differences: Chi² = 0.32, df = 1 (P = 0.57), I² = 0% | | | | | | | | | |

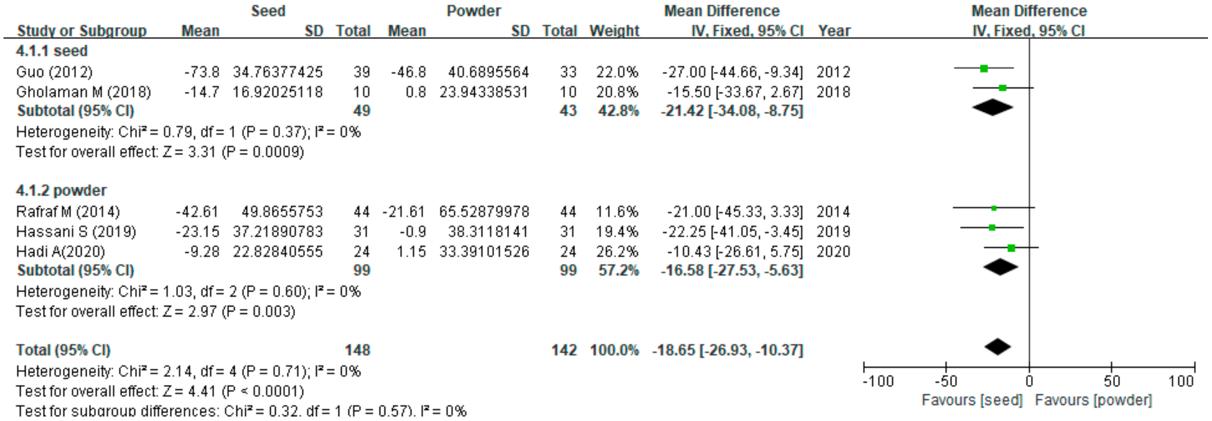


Figure S1. meta-analysis of seed form vs powder form. [31, 32, 34-36] ■ the position of the square represents the risk ratio, while the size of the square shows the weight of the study; the black line indicates the 95% confidence interval of the result; ◆ the diamond depicts overall pooled effect from the included studies.

| Study or Subgroup | Fenugreek Mean | Fenugreek SD | Fenugreek Total | Placebo Mean | Placebo SD | Placebo Total | Weight | Mean Difference IV, Fixed, 95% CI |
|---|---|---|---|---|---|---|---|---|
| **5.1.1 Hydro-alcoholic extracts** | | | | | | | | |
| Pickering E. (2023) | -7.7 | 29.59189 | 25 | 6.3 | 18.59355 | 23 | 6.1% | -14.00 [-27.87, -0.13] |
| **Subtotal (95% CI)** | | | **25** | | | **23** | **6.1%** | **-14.00 [-27.87, -0.13]** |
| Heterogeneity: Not applicable | | | | | | | | |
| Test for overall effect: Z = 1.98 (P = 0.05) | | | | | | | | |
| **5.1.2 Sapponin** | | | | | | | | |
| LU (2008) | -33.12 | 28.40409125 | 46 | -1.26 | 28.04536325 | 23 | 5.9% | -31.86 [-45.96, -17.76] |
| Verma N (2016) | -33.26 | 24.88748079 | 77 | -11.15 | 34.9021475 | 77 | 12.7% | -22.11 [-31.68, -12.54] |
| **Subtotal (95% CI)** | | | **123** | | | **100** | **18.6%** | **-25.19 [-33.11, -17.27]** |
| Heterogeneity: Chi² = 1.26, df = 1 (P = 0.26); I² = 20% | | | | | | | | |
| Test for overall effect: Z = 6.23 (P < 0.00001) | | | | | | | | |
| **5.1.3 Galactomannan** | | | | | | | | |
| Rashid R (2019) | -26.1 | 9.56035564 | 32 | 4.14 | 6.12793603 | 32 | 75.3% | -30.24 [-34.17, -26.31] |
| **Subtotal (95% CI)** | | | **32** | | | **32** | **75.3%** | **-30.24 [-34.17, -26.31]** |
| Heterogeneity: Not applicable | | | | | | | | |
| Test for overall effect: Z = 15.06 (P < 0.00001) | | | | | | | | |
| **Total (95% CI)** | | | **180** | | | **155** | **100.0%** | **-28.32 [-31.73, -24.90]** |
| Heterogeneity: Chi² = 6.87, df = 3 (P = 0.08); I² = 56% | | | | | | | | |
| Test for overall effect: Z = 16.25 (P < 0.00001) | | | | | | | | |
| Test for subgroup differences: Chi² = 5.61, df = 2 (P = 0.06), I² = 64.4% | | | | | | | | |

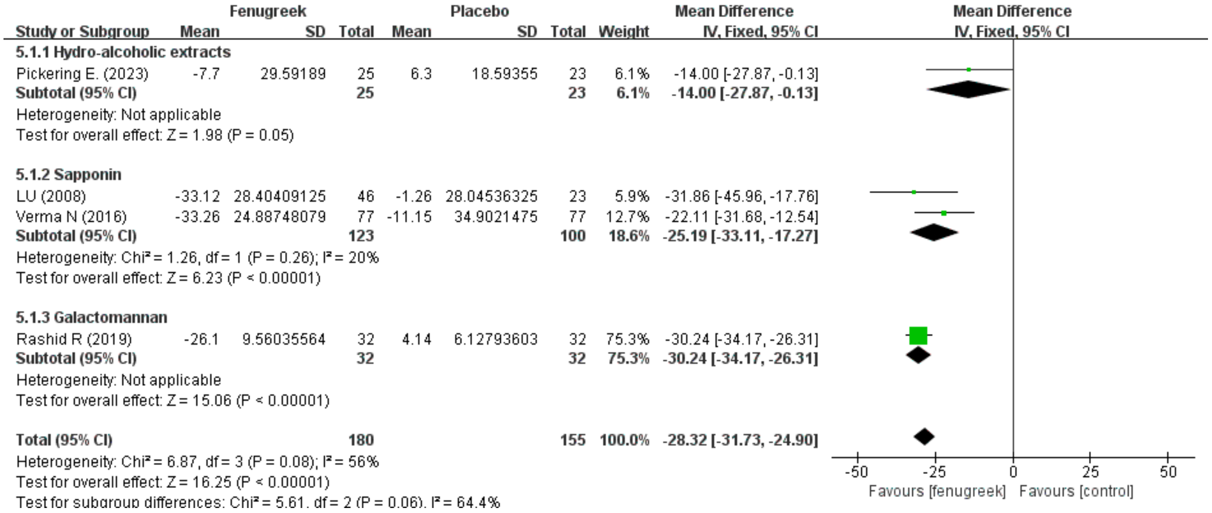


Figure S2. meta-analysis of hydroalcoholic extracts vs galactomannan vs saponin. [11, 28, 30, 33] ■ the position of the square represents the risk ratio, while the size of the square shows the weight of the study; the black line indicates the 95% confidence interval of the result; ◆ the diamond depicts overall pooled effect from the included studies.
